# Utilization of HIV-1 envelope V3 to identify X4- and R5-specific Tat and LTR sequence signatures

**DOI:** 10.1186/s12977-016-0266-9

**Published:** 2016-05-03

**Authors:** Gregory C. Antell, Will Dampier, Benjamas Aiamkitsumrit, Michael R. Nonnemacher, Jeffrey M. Jacobson, Vanessa Pirrone, Wen Zhong, Katherine Kercher, Shendra Passic, Jean W. Williams, Gregory Schwartz, Uri Hershberg, Fred C. Krebs, Brian Wigdahl

**Affiliations:** Department of Microbiology and Immunology, Drexel University College of Medicine, Philadelphia, PA USA; Center for Molecular Virology and Translational Neuroscience, Institute for Molecular Medicine and Infectious Disease, Drexel University College of Medicine, Philadelphia, PA USA; School of Biomedical Engineering, Science, and Health Systems, Drexel University, Philadelphia, PA USA; Division of Infectious Diseases and HIV Medicine, Department of Medicine, Drexel University College of Medicine, Philadelphia, PA USA; Center for Clinical and Translational Medicine, Institute for Molecular Medicine and Infectious Disease, Drexel University College of Medicine, Philadelphia, PA USA; Sidney Kimmel Cancer Center, Thomas Jefferson University, Philadelphia, PA USA

**Keywords:** HIV-1, Co-receptor, Tropism, LTR, Tat, V3, gp120, Diversity, Divergence, Transcription factor

## Abstract

**Background:**

HIV-1 entry is a receptor-mediated process directed by the interaction of the viral envelope with the host cell CD4 molecule and one of two co-receptors, CCR5 or CXCR4. The amino acid sequence of the third variable (V3) loop of the HIV-1 envelope is highly predictive of co-receptor utilization preference during entry, and machine learning predictive algorithms have been developed to characterize sequences as CCR5-utilizing (R5) or CXCR4-utilizing (X4). It was hypothesized that while the V3 loop is predominantly responsible for determining co-receptor binding, additional components of the HIV-1 genome may contribute to overall viral tropism and display sequence signatures associated with co-receptor utilization.

**Results:**

The accessory protein Tat and the HlV-1 long terminal repeat (LTR) were analyzed with respect to genetic diversity and compared by Jensen–Shannon divergence which resulted in a correlation with both mean genetic diversity as well as the absolute difference in genetic diversity between R5- and X4-genome specific trends. As expected, the V3 domain of the gp120 protein was enriched with statistically divergent positions. Statistically divergent positions were also identified in Tat amino acid sequences within the transactivation and TAR-binding domains, and in nucleotide positions throughout the LTR. We further analyzed LTR sequences for putative transcription factor binding sites using the JASPAR transcription factor binding profile database and found several putative differences in transcription factor binding sites between R5 and X4 HIV-1 genomes, specifically identifying the C/EBP sites I and II, and Sp site III to differ with respect to sequence configuration for R5 and X4 LTRs.

**Conclusion:**

These observations support the hypothesis that co-receptor utilization coincides with specific genetic signatures in HIV-1 Tat and the LTR, likely due to differing transcriptional regulatory mechanisms and selective pressures applied within specific cellular targets during the course of productive HIV-1 infection.

## Background

HIV-1 entry is a receptor-mediated, pH-independent process occurring via the direct interaction between viral envelope glycoprotein (gp)120 and the host cell CD4 receptor molecule, as well as one of the two most commonly encountered co-receptor molecules, CCR5 or CXCR4 [1]. The HIV-1 gp120 entry protein consists of five variable regions (V1–V5), which are highly modified by insertion, deletion, and substitution mutations, interspersed among five constant regions (C1–C5). Regardless of this sequence hypervariability, the overall structure and function of gp120 is highly conserved. Of particular importance to the HIV-1 entry mechanism is the third variable loop (V3), which has been shown to consist of 34–36 amino acid residues (most commonly 35 residues). The V3 domain has been extensively studied as it has been identified as the principal neutralizing domain (PND) on the viral envelope gp120 [2–4] and the viral partner in selective interactions with the different co-receptors [5].

The V3 region is a major determinant in predicting HIV-1 entry phenotype, distinguishing non-syncytium inducing (NSI) from syncytium inducing (SI) strains and macrophage tropic from non-macrophage tropic strains [5–8]. In this regard, it was discovered that a T cell tropic (T-tropic) isolate, or SI virus, preferentially uses the co-receptor CXCR4, and has consequently been referred to as an X4 virus. In contrast, a primary macrophage tropic (M-tropic) isolate, or NSI virus, was found to preferentially use the co-receptor CCR5 for entry and therefore referred to as an R5 virus [9, 10]. Previously, viral tropism based on co-receptor usage was used interchangeably with tropism defined by cellular target; however, several studies have shown that while co-receptor usage can at times be linked with cellular tropism, it is important to discuss viral infection in terms of utilization of a co-receptor in conjunction with the phenotype of the target cell [11, 12]. As an example, recent investigations have shown that HIV-1 infectious molecular clones containing transmitted/founder (T/F) genome sequences preferentially utilized CCR5 as a co-receptor during entry and were able to replicate efficiently in primary CD4+ T cells [13, 14]. These molecular clones also exhibited reduced replication efficiency in monocyte-derived macrophages (MDMs), in contrast to the prototypic M-tropic strains of HIV-1 [13].

For high-throughput applications, co-receptor utilization predictions can be performed on Env-V3 sequences computationally [15, 16]. To this end, the internet-based bioinformatic method, position-specific scoring matrices (Web-PSSM), utilizes sequences of known entry phenotype to determine if an Env-V3 sequence is CCR5- or CXCR4-utilizing (R5 or X4 Env-V3 sequences). This algorithm indicates the propensity of the virus to utilize CXCR4 at both high sensitivity (84 %) and specificity (96 %), with X4 virus sequences exhibiting high scores and R5 sequences exhibiting low scores, while intermediate Web-PSSM scores indicates both R5 and X4 virus sequences as well as dual tropic X4/R5 virus sequences [17]. Typically, X4 viruses emerge gradually in a subset of patients due to accumulation of amino acid changes within the V3 loop, particularly at positions 11 and 25. While HIV-1 co-receptor usage has been demonstrated to be directly associated with the genotype of V3 loop, as described above, other amino acid residues within gp120 V1, V2, C4, and a number of regions of gp41, have also been associated with co-receptor usage [18–27]. As conformational changes within the V3 loop occur upon the binding of gp120 and CD4, it is possible that the co-receptor usage-associated amino acid residues within other regions of envelope participate in the structural rearrangement of gp120 [28].

The overall goal of this study was to identify and characterize genetic differences between CCR5- and CXCR4-utilizing HIV-1 sequences beyond the V3 loop of envelope as defined by genotypic prediction. Specifically, HIV-1 gp120 and Tat amino acid sequences and the HIV-1 long terminal repeat (LTR) nucleotide sequences were selected for this analysis. The HIV-1 Tat protein derives its name from the fact that its primary function during viral replication is to serve as the trans-activator of transcription. While not directly involved in HIV-1 entry, Tat has a multitude of intracellular host binding partners and functions. The HIV-1 LTR contains an abundance of transcription factor binding sites upstream of the transcription start site that alter levels of HIV-1 transcription, often in a cell type-dependent manner. Importantly, these binding sites can work independently, in concert, or antagonistically, with a single nucleotide polymorphism capable of producing dramatic changes in binding, including the complete abrogation of transcription factor binding [29, 30].

We hypothesized that co-evolved R5- or X4-associated genetic signatures emerge in viral genes and proteins that are not directly associated with entry, and suggest that these differences are reflective of evolutionary constraints applied by different cellular milieus that associate, co-evolve, or co-adapt with co-receptor usage and may collectively guide tissue- and/or cell type-specific replication patterns, as well as organ-specific disease pathogenesis. The validity of this hypothesis is supported by the association of X4 virus with depleted CD4+ T-cell levels and enhanced disease progression, as well as the tendency of R5 virus to infect cells of the monocyte-macrophage lineage and to be found at the time of transmission [31]. However, the full extent of the underlying changes in the viral genome that could produce such a shift remains unknown.

To remedy this, we have taken a genetic approach with functional underpinnings that have centered on dividing HIV-1 co-linear sequences (gp120, Tat, and LTR) into two co-receptor utilization groups using genotypic prediction methods. Subsequently, we used these two groups of sequences to explore the differences in the remainder of gp120 sequences outside of Env-V3 as well as co-linear Tat and LTR sequences (Table 1). Sequence alignments of each co-receptor usage-defined population of co-linear gp120, Tat, or LTR sequences were quantitatively evaluated at each amino acid (gp120 and Tat) or nucleotide (LTR) position utilizing first-order diversity and Jensen–Shannon divergence. Together, diversity and divergence provide metrics to characterize the position-specific variation of amino acids or nucleotides, as well as provide a quantitative method to compare this information between R5- and X4-defined sequences. This strategy has provided a straightforward genetic approach to define specific sequences in Tat and the LTR, or potentially any other HIV-1 sequence, that are co-selected with CXCR4- and CCR5-dependent entry.

**Table 1:** Identification of HIV-1 Tat and LTR sequences co-linear to CCR5- and CXCR4-utilizing Env-V3 sequences defined by Web-PSSM scoring

Genomic region	CCR5	CXCR4
gp120	1678	52
Tat	504	31
LTR	615	35

HIV-1 amino acid sequences for gp120 and Tat and nucleotide sequences for LTR were classified as being derived from CCR5- or CXCR4-utilizing genomes according to Web-PSSM prediction scores utilizing the Env-V3 sequence. Sequences with intermediate scores PSSM scores are derived from R5, X4, or dual-tropic X4/R5 viruses and were not included in further analysis

## Results and discussion

Given the goal of this research was to determine if genetic signatures co-evolved between different regions of the HIV genome, it was first necessary to find patients that had sequences from the genomic areas of interest. From the Los Alamos National Library (LANL) database, subtype B sequences derived from patient samples were downloaded and aligned to the HXB2 genome. In total, more than 2500 samples were isolated, which included a full V3 of 35 amino-acids and at least one other co-linear sequence in the Tat or LTR regions. This also included 1730 full gp120 sequences. Table 1 shows the breakdown of sequences for each region and the selection criteria are further described in the Methods. For all results discussed below, we have analyzed the diversity/divergence of the amino acid sequences of gp120 and Tat proteins while the nucleotides of the LTR have been analyzed.

### Genetic diversity is correlated in R5- and X4-classified HIV-1 gp120, Tat, and LTR sequence populations

**Fig. 1:**
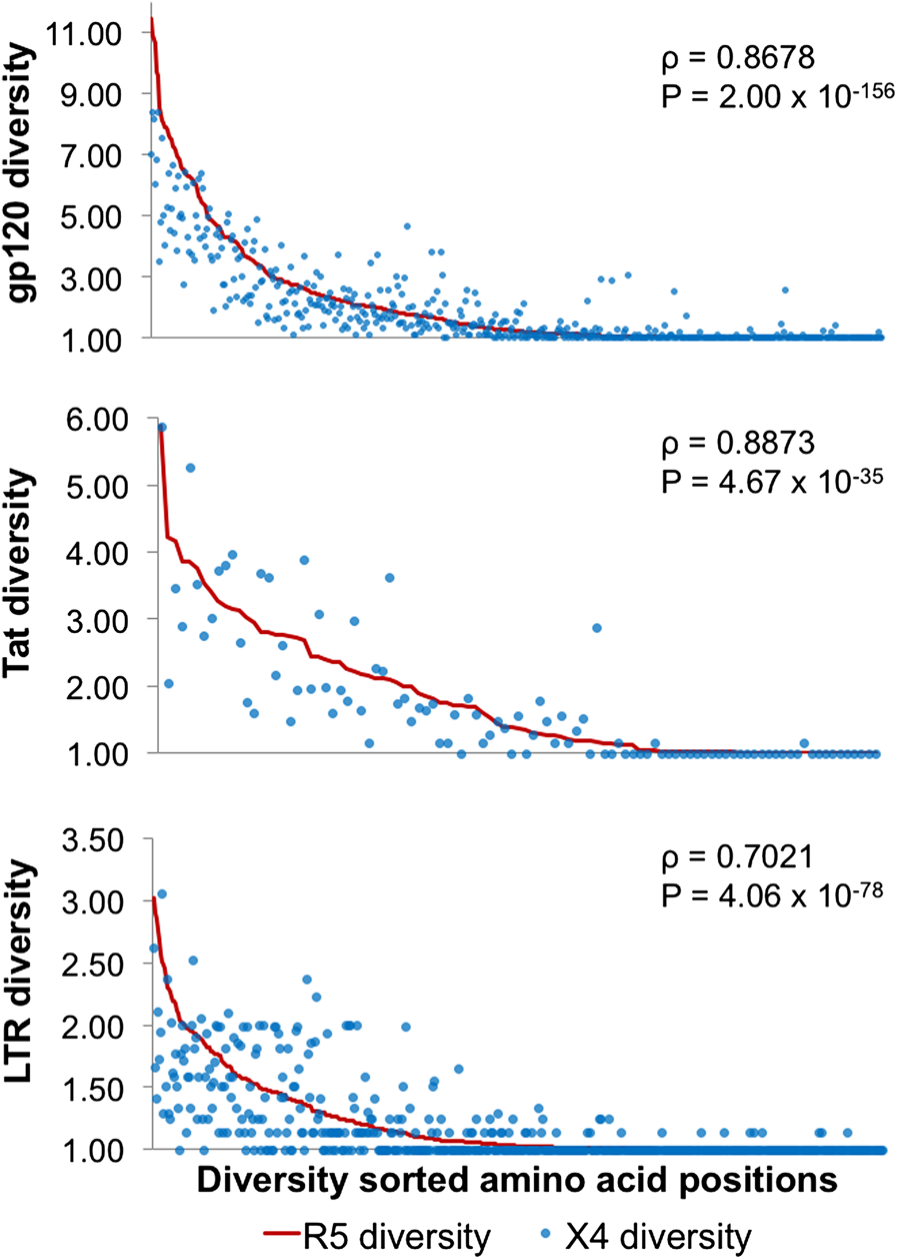
HIV-1 genetic diversity is highly correlated between corresponding positions in R5- and X4-classified gp120, Tat, and LTR sequence populations. The genetic diversity (order = 1) of each position of gp120, Tat, and LTR was calculated according to Eq. 1. The positions were sorted across the *x-axis* according to the R5 diversity values (*red line*), with the corresponding X4 positions plotted (*blue dots*). With this visualization, the vertical distance between the *line* and the *corresponding dot* represents the difference in diversity between the R5- and X4-classified sequences at each position. In general, the X4 values were found to cluster around the R5 values, with a slight skew towards less diversity within the X4 population. Spearman’s rank correlation was performed to assess the correlation between R5 and X4 diversity for gp120 (ρ = 0.8678, P = 2.00 × 10^−156^), Tat (ρ = 0.8873, P = 4.67 × 10^−35^), and LTR (ρ = 0.7021, P = 4.06 × 10^−78^). In all cases, R5 and X4 diversity were well-correlated

Spearman’s rank correlation was performed to assess the correlation between R5 and X4 diversity for gp120 (ρ = 0.8678, P = 2.00 × 10^−156^), Tat (ρ = 0.8873, P = 4.67 × 10^−35^), and LTR (ρ = 0.7021, P = 4.06 × 10^−78^) (Fig. 1). In all cases, R5 and X4 diversity were well-correlated, with the P value indicating support for the alternative hypothesis that X4 and R5 diversity is unrelated. Because first-order diversity was utilized in this analysis rather than richness (order = 0), and further supported by rarefaction analysis of the sample sizes, it is unlikely that differences in diversity are a reflection of the differences in sample size between the R5 and X4 sequence groups. This result indicates that, in general, corresponding amino acid (gp120 and Tat) or nucleotide (LTR) positions are similarly constrained in their usage with respect to R5 and X4 sequences.

### Jensen–Shannon divergence correlates with differences in diversity in HIV-1 gp120, Tat, and LTR sequences

**Fig. 2:**
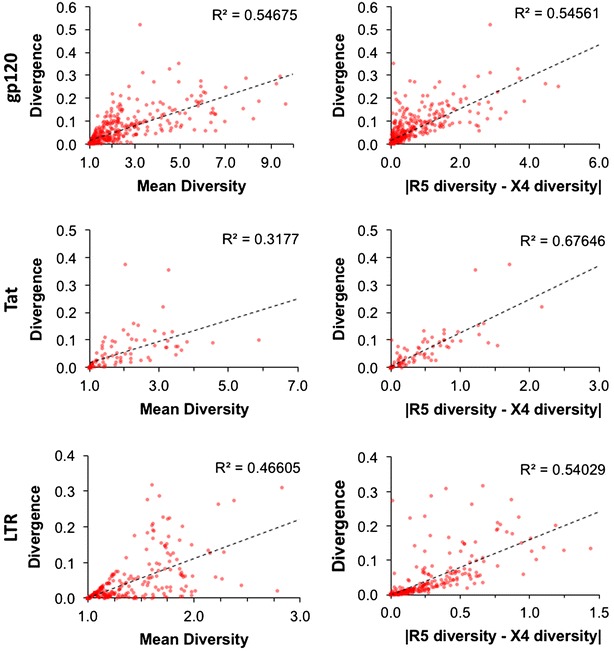
Jensen–Shannon divergence is correlated with both mean genetic diversity and the absolute difference in genetic diversity. The relationship between Jensen–Shannon divergence and genetic diversity (order = 1) in HIV-1 gp120, Tat, and LTR sequences was evaluated using Spearman’s rank correlation. Both the mean diversity of R5- and X4-classified sequences and the absolute difference between R5 and X4 diversity correlated with Jensen–Shannon divergence. This result indicates that large divergence can be a reflection of not only increased amounts of information (as indicated by high mean diversity), but also by the loss of information in one of the two groups (as indicated by the absolute difference in mean diversity)

Notably, Jensen–Shannon divergence correlates well with both mean diversity (ρ = 0.9226, 0.8552, and 0.9295 for gp120, Tat, and LTR, respectively) and the absolute difference in diversity (ρ = 0.9005, 0.8852, and 0.9685 for gp120, Tat, and LTR, respectively) (Fig. 2). Together, these observations indicate that the gain or loss of diversity in one of the populations is closely associated with high Jensen–Shannon divergence.

### Amino acid diversity and Jensen–Shannon divergence identified domains in gp120 responsible for CCR5 or CXCR4 co-receptor utilization

**Fig. 3:**
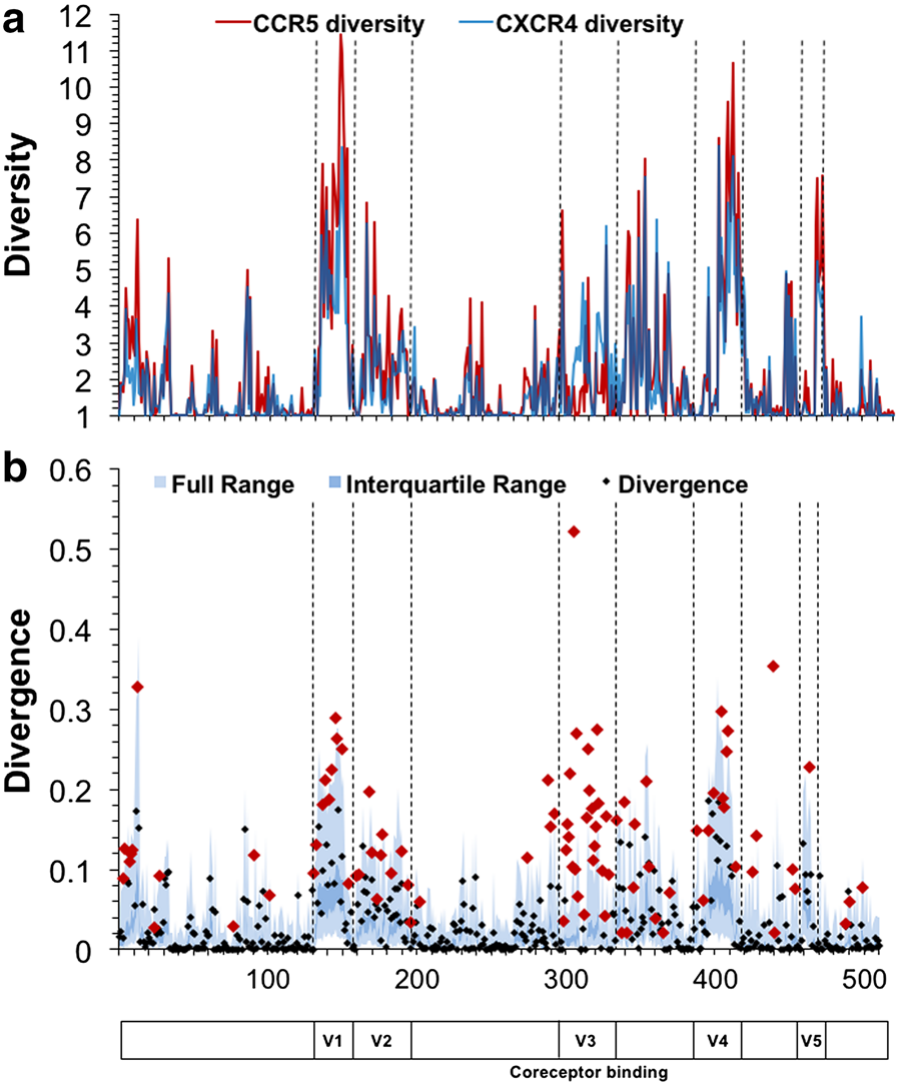
HIV-1 gp120 demonstrates high Jensen–Shannon divergence in regions with high genetic diversity. HIV-1 gp120 sequences were classified as CCR5 (R5) (n = 1681) or CXCR4 (X4) (n = 52) according to the predicted co-receptor usage of the V3 domain Web-PSSM score [17]. **a** The diversity index at a Hill number of 1 was calculated for each position for both R5 (*red*) and X4 (*blue*) gp120 amino acid sequence populations. Diversity values range from 1 to greater than 10, with the variable domains of gp120 displaying the greatest diversity. **b** The Jensen–Shannon divergence between R5 and X4 gp120 sequence populations was computed for each amino acid position and plotted with a *diamond*. Statistically divergent positions (P < 0.01) were plotted in *red*. A Monte Carlo permutation test was performed to iteratively group gp120 sequences into random groups and calculate a distribution of expected Jensen–Shannon divergence values. The full range of this distribution was plotted in *light blue* with the interquartile range plotted in *dark blue*. The full range of divergence for randomly generated groups is in close agreement with the combined diversity of the R5 and X4 populations

**Fig. 4:**
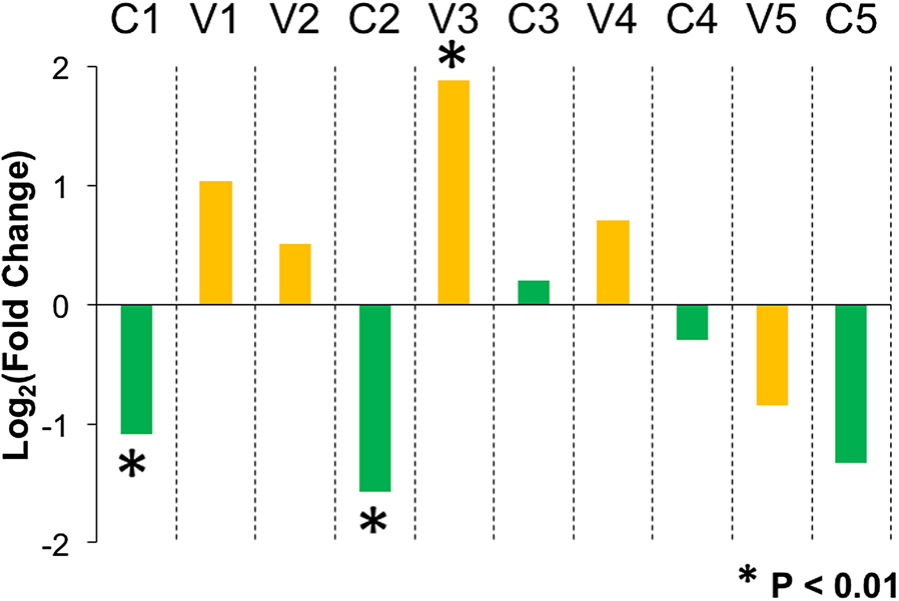
V3 domain of gp120 is enriched with statistically divergent positions. The 10 conserved and variable domains of gp120 were evaluated to determine if any regions were enriched in statistically divergent sites. A hypergeometric test was used to determine enrichment and depletion of statistically divergent positions, using the null hypothesis of equal distribution amongst domains. The V3 loop was identified as being highly enriched (P = 1.74 × 10^−11^), while the C1 domain (P = 3.03 × 10^−4^) and C2 domain (P = 1.28 × 10^−4^) were statistically depleted at P < 0.01 using a Benjamini–Hochberg multiple testing correction

The HIV-1 envelope protein gp120 was evaluated to detect the effectiveness of first-order sequence position diversity and Jensen–Shannon divergence with respect to identifying genetic signatures of co-receptor utilization, with the expectation that the V3 domain exhibits higher Jensen–Shannon divergence between R5- and X4-associated gp120 sequences. Diversity analysis, which as stated above was highly correlated between R5 (red) and X4 (blue) sequence populations, confirmed that the variable loops of gp120 display the greatest amount of amino acid diversity (Fig. 3a). The V1 and V4 variable domains, in particular, contain several positions that display diversity of greater than 10 at order = 1, with a large proportion of positions having a diversity >3. Calculation of Jensen–Shannon divergence between the R5 and X4 populations identified 92 statistically significant positions throughout gp120 (Fig. 3b). A hypergeometric statistical test was used to determine if any domains of gp120 were enriched in statistically divergent positions, as opposed to the null hypothesis of equal distribution. Overall, the variable domains were enriched in divergent positions when compared to the null model, while the conserved domains were depleted, although the C3 and V5 domains were in slight opposition to this trend (Fig. 4). Specifically, the V3 loop was very highly enriched [log_2_(fold change) = 1.89, P = 1.74 × 10^−11^] while the C1 domain [log_2_(fold change) = −1.09, P = 3.03 × 10^−4^] and C2 domain [log_2_(fold change) = −1.57, P = 1.28 × 10^−4^] were statistically depleted at P < 0.01 using a Benjamini–Hochberg multiple testing correction. With the understanding that gp120 and V3 behaved as expected following the application of diversity and divergence in this study, Tat and the LTR were investigated for similar signatures that may co-evolve with alterations in co-receptor utilization patterns exhibited by Env-V3.

### X4 Tat sequences demonstrate purifying selection in amino acid usage

**Fig. 5:**
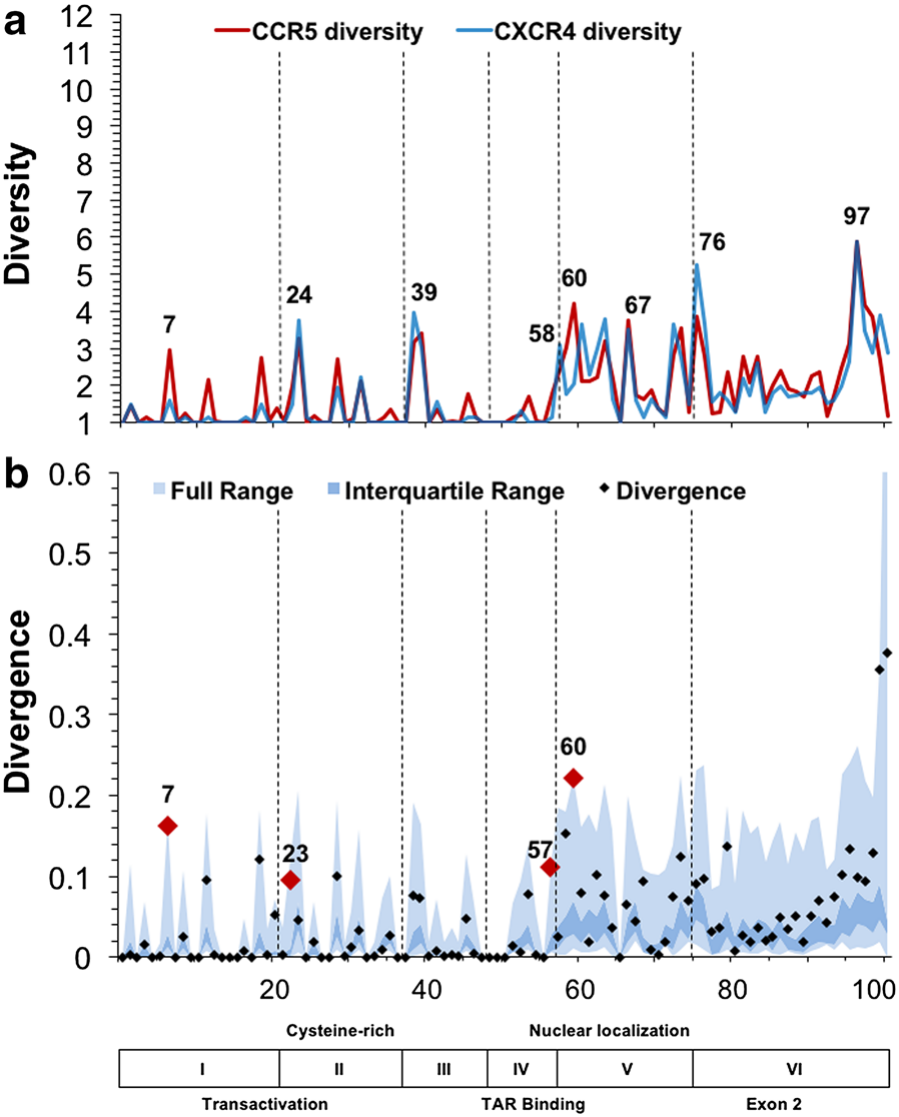
Jensen–Shannon divergence identifies positions of differential amino acid usage between R5 and X4 HIV-1 Tat sequences. HIV-1 Tat sequences were sorted into R5 (n = 504) and X4 (n = 31) populations according to the predicted co-receptor usage of the co-linear V3 domain as determined by Web-PSSM score. **a** The diversity index at order = 1 was calculated for each position for both R5 (*red*) and X4 (*blue*) Tat sequence populations. The diversity index between R5 and X4 populations displayed high similarity at nearly all positions, with the second half of Tat displaying higher diversity values overall for both populations. **b** The Jensen–Shannon divergence between R5 and X4 Tat sequences was computed for each amino acid position and plotted with a *diamond*. Statistically divergent positions 7, 23, 57, and 60 (P < 0.01) were plotted in *red* and consensus changes, positions 40 and 67, were plotted in *yellow*. A Monte Carlo permutation test was performed to iteratively group Tat sequences into random groups and calculate a distribution of expected Jensen–Shannon divergence values. The full range of this distribution was plotted in *light blue* with the interquartile range plotted in *dark blue*

**Fig. 6:**
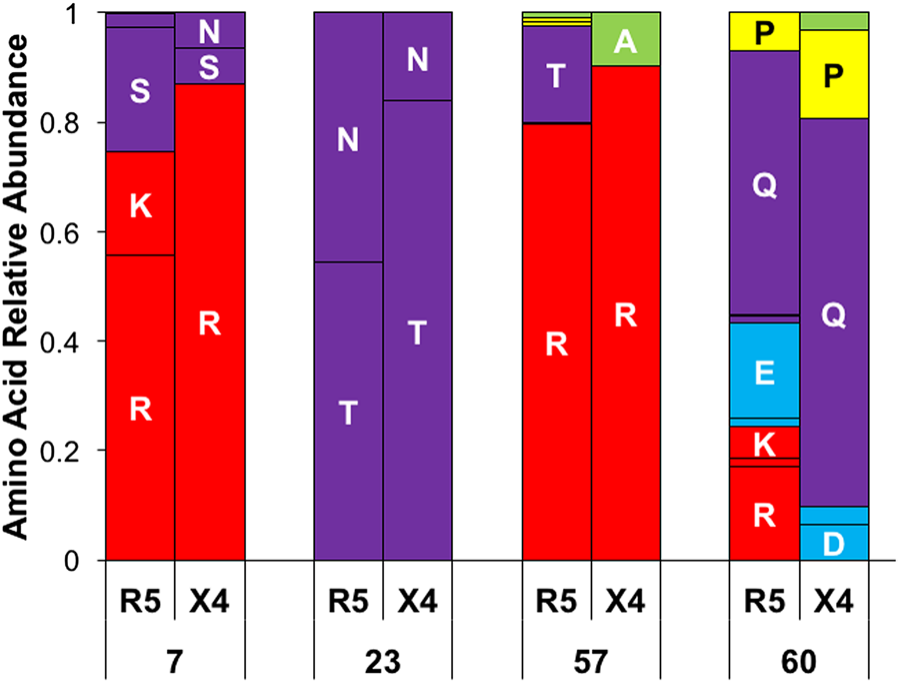
Statistically divergent positions between X4 and R5 HIV-1 Tat interchange amino acids for those with similar physiochemical properties. The amino acid usage in four HIV-1 Tat amino acid positions (7, 23, 57, and 60) was plotted for both R5 and X4 groups as a stacked bar chart representing the total genetic variation within each population at the respective positions. Amino acids were color coded according to physiochemical property using the following scheme: positively charged (*red*), negatively charged (*blue*), polar uncharged (*purple*), hydrophobic (*green*), and unclassified (glycine, proline, and cysteine, *yellow*). The amino acid positions 7, 23, 57, and 60 were selected due to their statistically significant Jensen–Shannon divergence

**Fig. 7:**
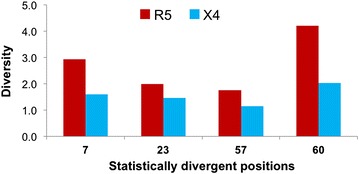
Statistically divergent Tat positions demonstrate reduced diversity within X4-classified sequences. Within HIV-1 Tat, four amino acid positions were identified as having statistically significant Jensen–Shannon divergence: 7, 23, 57, and 60. In all four cases, it was noted that X4-classified variants exhibited a lower overall genetic diversity at an order of 1, largely due to the enhanced presence of the most common variant in the X4 population. This pattern of diminished diversity within X4 in comparison to R5 suggests that a purifying selective force may be present, affecting a subset of HIV-1 Tat variants

In general, we observed an overall trend for Tat to have higher amino acid diversity in the fifth and sixth domains of both groups (Fig. 5a). When we considered Jensen–Shannon divergence, positions 7, 23, 57, and 60 were found to be statistically divergent and P < 0.01 when accounting for multiple testing with the Benjamini–Hochberg procedure (Fig. 5b). For all four divergent positions, the set of amino acids used in each population was similar, both with regard to the amino acids observed as well as the physiochemical properties of these amino acids (Fig. 6; Table 2). In all cases, the amino acid diversity of the X4 population was less than the diversity of the R5 population, demonstrating the qualitative trend that a subset of major variants become further enriched within the X4 population (Fig. 7). While there was no adequate statistical methodology to test the significance of the difference in diversity at a single position, a Fisher’s exact test was used to demonstrate the statistical enrichment of the consensus variants R7 (P = 0.00053), T23 (P = 0.0012), and Q60 (P = 0.0158) within the X4 group that is not a reflection of differences in R5 and X4 group sizes. In contrast, Tat variant R57 was not statistically enriched in the X4 population (P = 0.17). Regardless, this observation lends support to a mechanism in which the HIV-1 Tat X4 genotype undergoes purifying selection in concert with the change in V3 co-receptor usage from R5 to X4, whereas R5 Tat may be able to persist within a less constrained sequence space that allows it to effectively drive HIV-1 transcription in both T cells and MDMs and perhaps other cell lineages.

**Table 2:** HIV-1 R5 and X4 Tat amino acid usage

Position	AA	R5 count	R5 %	X4 count	X4 %
7	K	95	0.19	0	0.00
	N	12	0.02	2	0.06
	S	115	0.23	2	0.06
	R	281	0.56	27	0.87
23	N	230	0.46	5	0.16
	T	274	0.54	26	0.84
57	A	5	0.01	3	0.10
	R	400	0.79	28	0.90
	T	89	0.18	0	0.00
60	E	87	0.17	1	0.03
	D	7	0.01	2	0.06
	K	30	0.06	0	0.00
	Q	242	0.48	22	0.71
	P	35	0.07	5	0.16
	R	86	0.17	0	0.00

The raw counts and relative abundance value of amino acids present in statistically divergent Tat positions for both the R5 and X4 sequence groups (7, 23, 57 and 60)

The four Tat amino acid positions of interest are located within domains responsible for transactivation, cysteine-rich, TAR binding, and nuclear localization, respectively (Fig. 5). In order to further evaluate the amino acid usage of these positions, the relative abundance of each variant was plotted (Fig. 6). In both R5 and X4 Tat, arginine is the most common variant at position 7, although R7 is much more conserved within the X4 population. Previous studies have shown that an R7G substitution has only 93.9 ± 6.5 % of the transactivation activity of R7 [32]. While this effect size is small, it may contribute to differences in the transactivation activity between R5 and X4 HIV-1. Position 23 of Tat also is likely to play an important role in robust transactivation. In our data set, threonine was the most common variant observed at position 23, although asparagine was a frequent variant in both the R5 (46 %) and X4 (16 %) sequences. The T23N substitution has been shown to increase transactivation of the HIV-1 LTR, as well as binding to P-TEFb [33]. Accordingly, Tat N23 has been suggested to confer an advantage to HIV-1 by compensating for deleterious Tat mutations and supporting the replication of less fit drug-resistant or immune-evasive quasispecies [33]. Furthermore, the enrichment of the T23N substitution in the R5 Tat population may relate to the ability of R5 HIV-1 to productively infect both T cells and monocytes and the selection of R5 HIV-1 at the time of transmission, as opposed to X4 HIV-1 which lacks a strong association with monocyte infection and is selected against at the time of transmission. The co-selection and possible synergistic effect of these Tat variants remains an area of future investigation.

### Predicted transcription factor binding sites have statistically different binding affinity scores between X4 and R5 LTR sequences

**Fig. 8:**
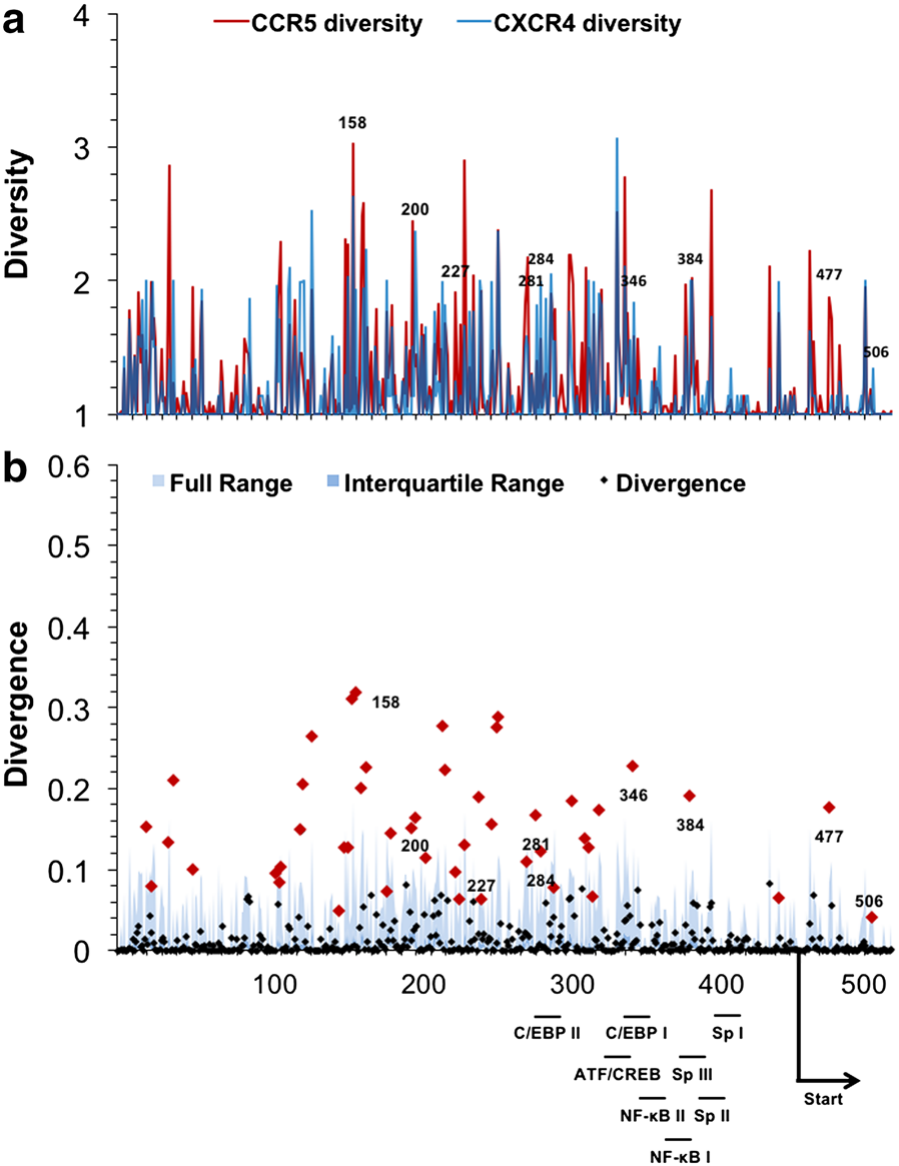
HIV-1 LTR demonstrates high divergence both upstream and downstream of the transcription start site. HIV-1 long terminal repeat (LTR) sequences were sorted into R5 (n = 615) and X4 (n = 35) populations according to the predicted co-receptor usage of the co-linear V3 region. **a** The diversity index at order = 1 was calculated for each position for both R5 (*red*) and X4 (*blue*) LTR sequence populations, numbered according to the HXB2 reference sequence. **b** Following the same approach applied for amino acid analysis, Jensen–Shannon divergence between R5 and X4 LTR sequences was computed for each nucleotide position and plotted. Statistically divergent positions were plotted in *red* and identified throughout the LTR, both upstream and downstream of the transcriptional start site and within transcription factor binding sites. A Monte Carlo permutation simulation was performed to randomly group LTR sequences and calculate a distribution of expected Jensen–Shannon divergence values, with the full range (*light blue*) and interquartile range (*dark blue*) of the distribution plotted across each position of the LTR

Genetic diversity and Jensen–Shannon divergence analyses were performed on LTR nucleotide sequences. 518 nucleotide positions spanning the HIV-1 U3, R, and U5 regions were evaluated. High levels of nucleotide diversity were present throughout the entire LTR and did not display a general pattern beyond being low, i.e. more highly conserved, in the approximately 50 nucleotides immediately downstream of the transcription start site that correspond to the TAR region of the LTR. This observation translated to large numbers of statistically significant Jensen–Shannon divergence scores (n = 48) between the two populations (Fig. 8). A number of these statistically divergent positions were identified at nucleotide positions within the core enhancer domain, the region of the LTR spanning approximately 200 nucleotides upstream of the transcription start site, while a high number of divergent positions were also identified in the less well characterized modulatory domain further upstream. Due to the high concentration of known transcription factor binding sites within the core enhancer domain, this region of the LTR was the focus of further analysis.

Binding of cellular transcription factors to the LTR has been shown to be one of the most critical parts of the viral life cycle with respect to acute infection but also in controlling the initial phases of genomic activation from latency. Throughout the HIV-1 LTR, eight well-known transcription factor binding sites were evaluated to determine if differential nucleotide usage exists between R5 and X4 populations: C/EBP-II (HXB2 positions 281–289), ATF-CREB (330–337), C/EBP-I (338–349), NF-κB-II (350–359), NF-κB-I (363–373), Sp-III (377–386), Sp-II (388–398), and Sp-I (399–408). Additionally, the TAR stem-loop region (HXB2 positions 454–518) was similarly investigated for R5- and X4-associated differences.

**Fig. 9:**
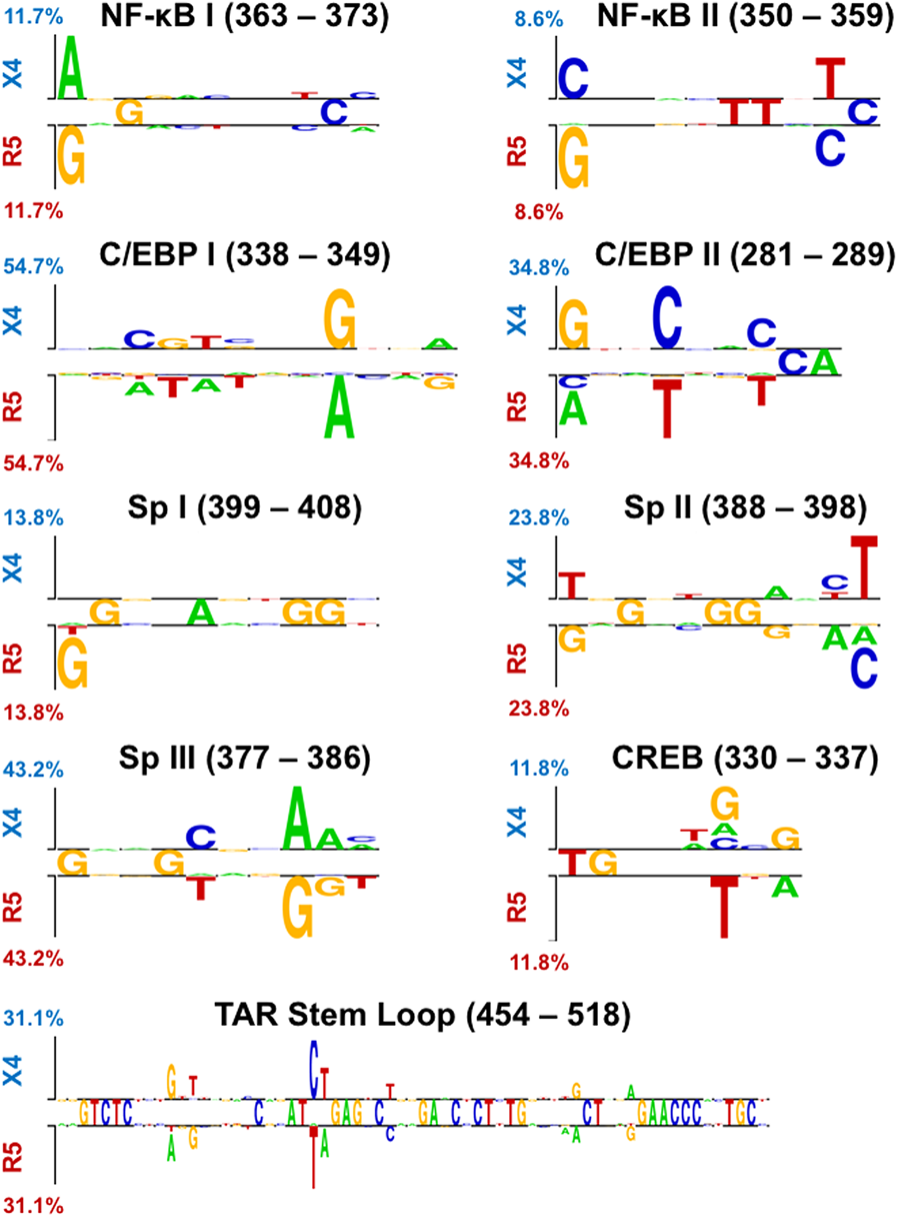
R5 and X4 LTR sequences demonstrate signature enriched nucleotide variants in transcription factor binding. HIV-1 transcription factors that have been confirmed in vitro, C/EBP-II (HXB2 positions 281–289), ATF-CREB (330–337), C/EBP-I (338–349), NF-κB-II (350–359), NF-κB-I (363–373), Sp-III (377–386), Sp-II (388–398), and Sp-I (399–408), as well as the TAR stem loop (454–518), were evaluated to detect enrichment and depletion of nucleotide variants in R5 and X4 sets of aligned LTR sequences using two sample logos. Enriched nucleotides were plotted proportional to the difference between the populations, with the sum of the most differential position plotted on the *vertical axis*

The difference in nucleotide usage within known transcription factor binding sites was visualized using two-sequence logos (Fig. 9). This visualization creates a sequence logo for each transcription factor binding site that indicates nucleotides that are enriched within either the R5 or X4 populations, and scaled according to the maximum difference in relative abundance, such that nucleotides more frequently found in the R5 or X4 population are displayed on the bottom or top partition of the two-sequence logo, respectively, while completely conserved nucleotides are displayed in the middle. This analysis demonstrated that the greatest relative abundance differences in nucleotide usage occurred within sites C/EBP-I (54.7 %), C/EBP-II (34.8 %), and Sp-III (43.2 %), as well as in the TAR stem loop (31.1 %). In contrast, NF-κB-I (11.7 %), NF-κB-II (8.6 %), Sp-I (13.8 %), Sp-II (23.8 %), and ATF-CREB (11.8 %) showed rather modest differences between X4 and R5 in terms of nucleotide usage. Statistically divergent positions were identified within several of these transcription factor binding sites (Fig. 8), specifically sites C/EBP-I (position 346), C/EBP-II (positions 281 and 284), and Sp-III (position 384), as well as position 477 of the TAR stem loop, in agreement with the maximal differences observed in the two-sequence logos. Specifically, when comparing R5 to X4 sequences, the aforementioned positions demonstrated a propensity for an A-to-G (HXB2 position 346) mutation within C/EBP-I, an A/C-to-G (HXB2 position 281) and a T-to-C (HXB2 position 284) mutation within C/EBP-II, and a G-to-A (HXB2 position 384) mutation within Sp-III. Finally, a large T-to-C (HXB2 position 477) mutation was observed within the bulge region of the TAR stem loop. The bulge region plays a crucial role in Tat recruitment and binding to the transcription complex, raising the possibility that X4 HIV-1 may contain a large subpopulation of genomes that have altered Tat recruitment and binding relative to R5 HIV-1 [34].

Although R5- and X4-specific nucleotide positions were identified throughout the LTR, it was not clear if those changes would result in meaningful differences between R5 and X4 in terms of transcription factor binding affinity. In order to quantitatively evaluate the difference between R5 and X4 LTR sequence groups, analysis was performed using the JASPAR database, a collection of transcription factor DNA-binding preferences modeled as matrices [35, 36]. Each LTR sequence was scanned for transcription factor binding sites by scoring against position weight matrices (PWMs), which were converted from JASPAR position frequency matrices (PFMs) downloaded from the JASPAR vertebrate database for C/EBP, SP1, NF-κB, and CREB. Each score was then compared to the maximum possible score of the corresponding PWM in order to determine a percentile score. Only binding sites with a mean percentile score >0.30 in either the R5- or X4-classified LTR sequences were considered for statistical analysis (Table 3). The distribution of R5- and X4-binding scores was statistically compared using a Kolmogorov–Smirnov (KS) test to calculate a P value.

**Table 3:** Predicted transcription factor binding sites in the HIV-1 LTR

Transcription factor	JASPAR matrix	In vitro confirmed	HXB2 index	Strand direction	X4 mean score	R5 mean score	P value
C/EBP	MA0102.1-CEBPA		76	Reverse	4.55	4.06	0.3860
	MA0102.2-CEBPA		79	Reverse	4.94	4.41	0.3860
	MA0102.2-CEBPA		81	Forward	4.24	3.12	0.2304
	*MA0102.1*-*CEBPA*		*150*	*Reverse*	*5.16*	*3.39*	<*0.0001*
	*MA0102.2*-*CEBPA*		*153*	*Reverse*	*6.35*	*4.58*	<*0.0001*
	*MA0102.2*-*CEBPA*		*154*	*Forward*	*4.53*	*1.83*	<*0.0001*
	MA0102.1-CEBPA		197	Reverse	5.27	4.63	0.0092
	MA0102.2-CEBPA		200	Reverse	4.33	4.34	0.0063
	*MA0102.1*-*CEBPA*	*C/EBP II*	*278*	*Reverse*	*5.84*	*6.29*	*0.0005*
	*MA0102.2*-*CEBPA*	*C/EBP II*	*281*	*Reverse*	*5.26*	*5.70*	*0.0010*
	*MA0102.3*-*CEBPA*	*C/EBP II*	*281*	*Forward*	*7.65*	*9.67*	*0.0008*
	*MA0102.2*-*CEBPA*	*C/EBP I*	*342*	*Forward*	*5.26*	*4.65*	<*0.0001*
CREB	MA0018.2-CREB1		173	Forward	4.74	3.93	0.1020
	MA0018.2-CREB1	ATF/CREB	330	Forward	5.25	5.63	0.4547
	MA0018.2-CREB1		410	Reverse	4.43	4.68	0.8684
NF-kB	MA0105.1-NFKB1	NF-kB II	350	Forward	13.66	14.52	0.9605
	MA0105.1-NFKB1	NF-kB II	350	Reverse	7.43	8.30	0.9605
	MA0105.2-NFKB1	NF-kB II	350	Forward	6.67	7.41	0.9605
	MA0105.3-NFKB1	NF-kB II	350	Forward	12.37	13.41	0.9722
	MA0105.1-NFKB1	NF-kB II	351	Reverse	4.85	5.31	0.9813
	MA0105.1-NFKB1	NF-kB I	363	Forward	6.18	6.77	0.8062
	MA0105.2-NFKB1	NF-kB I	363	Forward	8.20	8.62	0.8062
	MA0105.2-NFKB1	NF-kB I	363	Reverse	7.60	7.88	0.8345
	MA0105.1-NFKB1	NF-kB I	364	Forward	14.55	14.35	1.0000
	MA0105.1-NFKB1	NF-kB I	364	Reverse	8.33	8.26	1.0000
	MA0105.2-NFKB1	NF-kB I	364	Forward	7.44	7.29	1.0000
	MA0105.3-NFKB1	NF-kB I	364	Forward	15.66	15.15	0.8907
	MA0105.1-NFKB1	NF-kB I	365	Reverse	5.34	5.15	1.0000
	MA0105.2-NFKB1	NF-kB	504	Forward	6.09	6.46	0.9850
	MA0105.2-NFKB1	NF-kB	504	Reverse	8.56	8.83	0.9881
	MA0105.1-NFKB1	NF-kB	505	Reverse	6.01	6.30	0.9850
Sp	MA0079.2-SP1		98	Reverse	4.68	5.68	0.0093
	MA0079.1-SP1		99	Forward	5.01	5.35	0.0093
	*MA0079.1*-*SP1*		*223*	*Forward*	*4.35*	*3.31*	<*0.0001*
	*MA0079.1*-*SP1*		*224*	*Forward*	*4.57*	*2.05*	<*0.0001*
	MA0079.1-SP1		266	Reverse	4.36	3.37	0.1034
	MA0079.2-SP1	Sp-III	373	Reverse	5.30	5.86	0.5696
	MA0079.1-SP1	Sp-III	374	Forward	4.12	4.82	0.0755
	*MA0079.2*-*SP1*	*Sp*-*III*	*376*	*Reverse*	*5.22*	*6.46*	<*0.0001*
	*MA0079.1*-*SP1*	*Sp*-*III*	*377*	*Forward*	*3.86*	*5.56*	<*0.0001*
	*MA0079.2*-*SP1*	*Sp*-*III*	*382*	*Reverse*	*4.71*	*5.81*	<*0.0001*
	MA0079.2-SP1	Sp-II	387	Reverse	6.72	7.11	0.7894
	MA0079.3-SP1	Sp-II	387	Reverse	10.11	10.46	0.6982
	MA0079.1-SP1	Sp-II	388	Forward	5.78	6.14	0.2648
	MA0079.2-SP1	Sp-II	392	Reverse	6.62	7.07	0.1179
	MA0079.2-SP1	Sp-II	393	Reverse	5.51	6.81	0.0168
	MA0079.1-SP1	Sp-I	398	Forward	4.35	4.35	0.6176
	MA0079.2-SP1	Sp-I	398	Reverse	6.69	7.21	0.5383
	MA0079.1-SP1	Sp-I	399	Forward	4.30	4.47	0.5539
	MA0079.2-SP1	Sp-I	400	Reverse	5.78	5.83	1.0000
	MA0079.1-SP1		479	Forward	5.21	5.02	0.9279

LTR sequences classified as either R5 or X4 based on their co-linear Env-V3 sequence were scanned for potential transcription factor binding sites. All binding sites with a percentile score >0.3 in either of the two groups were included in this analysis. The overall score distribution of R5 and X4 binding sites was compared using a KS-test, and multiple testing was accounted for using the Benjamini–Hochberg procedure. Statistically significant sites (P < 0.01) are highlighted in italics

Using the JASPAR matrices, we were able to correctly identify the locations of each of the eight known transcription factor binding sites within the LTR when using a percentile scoring threshold of 0.3. At this threshold, the JASPAR matrices for C/EBP, Sp1, and CREB also identified several other potential novel binding locations (Table 3). Statistical analysis identified differential binding scores between the R5 and X4 populations at sites C/EBP-I, C/EBP-II, and Sp-III, but not at known transcription factor binding sites Sp-I or Sp-II (Table 3). This result is in agreement with the positions identified using Jensen–Shannon divergence. Additionally, novel putative Sp1 and C/EBP binding sites with differential R5/X4 JAPSAR scores were noted at positions 223 and 150, respectively.

Interestingly, the relative magnitude of R5 and X4 mean binding scores of C/EBP-I and -II were opposite one another, with X4 LTRs having a greater mean binding score than R5 LTRs for C/EBP-I, whereas R5 LTRs have a greater mean binding score for C/EBP II. The novel putative C/EBP site followed the trend of C/EBP site I, and had a greater mean score among X4 LTRs. This relationship may be a compensatory effect by which the diminished binding affinity of C/EBP II, as the virus mutates from R5 to X4, leads to greater binding to C/EBP I and perhaps other putative C/EBP sites. This overall trend is also reflected among Sp binding sites. As the binding affinity of Sp-III diminishes in X4 virus when compared to R5, a putative novel Sp site at position 223 gains enhanced binding affinity as indicated by JASPAR scoring. Generally, Sp sites have been shown to be more important for LTR-driven transcription in T lymphocytes than cells of the monocyte-macrophage lineage [37]. Furthermore, transcription factor binding at Sp-III varies with respect to the level of differentiation of monocytes [38]. Overall, we find that LTR-driven transcription is modulated by proteins of the Sp family in a manner that is specific to cell phenotype. As an important contrast, CREB and NF-κB binding sites did not demonstrate a difference in overall binding affinity between the R5 and X4 groups, although the analysis identified all known binding sites in both R5 and X4 populations. This is likely due to the fact that these sites have been shown to be essential for both T-lymphocyte and monocyte-macrophage replication [39–42].

## Conclusion

The V3 domain of the HIV-1 *env* gene evolves throughout the course of infection, often resulting in a switch from an R5 to an X4 swarm. However, the characterization of R5 and X4 HIV-1 has not been defined beyond the envelope, specifically with respect to the transcriptional regulation of HIV-1. Our findings indicate that when comparing X4 HIV-1 to R5 HIV-1 sequences, Tat amino acids variants are more strictly selected at several key positions and specific LTR nucleotide variants are preferentially present in X4 HIV-1 sequences when compared to R5 HIV-1. One particular caveat of our analysis, and any that involves historical sequence review, is our choice of the functional annotation algorithm used in these studies. While the specific results may differ to some extent with the choice of computational tools used in a given study, the overall finding that there is co-evolution between gp120 and other regions of the HIV-1 genome remains consistent. These results are significant because they indicate that the transcriptional phenotype of HIV-1 may diverge with respect to co-receptor utilization. Importantly, the HIV-1 amino acid positions identified in Tat as different between X4 and R5 play roles in supporting robust transactivation, while the LTR nucleotide mutations associated with X4 and R5 strains are found within known and putative transcription factor binding sites and may affect their occupancy and contributions to the regulation of HIV-1 gene expression. We regard the genetic variation between X4 and R5 HIV-1 Tat and LTR sequences to be indicative of selection caused by the differential intracellular environments of cells preferentially infected by X4 versus R5 HIV-1 quasispecies. Consequently, the evolution of HIV-1 from an R5 to an X4 swarm likely requires adaptation at the level of transcriptional control in addition to co-receptor binding and entry.

## Methods

### HIV-1 sequence collection

HIV-1 sequences containing the Env-V3 region in addition to a co-linear Tat or LTR were collected and annotated from the Los Alamos National Laboratory (LANL) HIV Sequence Database as of October 2014, while additional sequences from the Drexel Medicine CNS Research and AIDS Eradication Study (CARES) Cohort were added to supplement the total number of sequences publicly available. The Drexel Medicine CARES Cohort is a subtype B patient cohort from Philadelphia, Pennsylvania and has been previously described [43–46]. The sequences from the Drexel Medicine CARES Cohort have been submitted to Genbank under BioProject ID: PRJNA319822. To reduce the effect of regional and subtype differences, the LANL database query was limited to include only subtype B sequences isolated from North America. The query was further limited to a single sequence per patient using the LANL query tool which specifically excludes laboratory strain sequences or those used for functional studies. Table 1 shows the breakdown of sequences for each region.

### Co-receptor usage classification

The in silico co-receptor usage prediction tool Web-PSSM was used to classify all sequences as CCR5- or CXCR4-utilizing based on the score of the co-linear Env-V3 amino acid sequence [17]. Numerous exclusion methods were utilized to reduce noise introduced by Web-PSSM predictions as discussed previously [47]. Sequences were excluded from the study if the V3 region was not 35 amino acid residues in length, if the V3 percentile determined by Web-PSSM was greater than 0.95 (indicating that a given sequence may not be a V3 sequence), or if the V3 PSSM score was in the ‘indeterminate range’ (using scoring cutoffs of >−2.88 and <−6.96 for X4 and R5 Env-V3 sequences, respectively), which was defined as a scoring range consisting of sequences with R5 and/or X4 properties including sequences that are dual tropic (X4/R5). Using these cutoffs, this predictor has an 84 % sensitivity and 96 % specificity indicating its ability to detect X4 binding sequences and non-binding sequences, respectively [17]. This filtering method allowed the genetic analysis to focus on sequences with the highest confidence classification in the PSSM-derived distribution, definitively signifying CCR5- or CXCR4-utilizing Env-V3 sequences. Following classification as R5 or X4, the co-linear gp120, Tat, and LTR sequences were aligned to the HXB2 reference sequence (K03455) using Multiple Sequence Comparison by Log-Expectation (MUSCLE), (version 5.05) [48] utilizing default parameters; insertions relative to the reference were removed to simplify the analysis. This pipeline resulted in R5- and X4-associated and multiple sequence alignments for each gp120, Tat, and LTR sequence (Table 1).

### Genetic diversity and rarefaction

The diversity of each amino acid or nucleotide position of the respective multiple sequence alignments was calculated using a window length, *w*, of 1 and an order of 1 [equivalent to exp(Shannon entropy with base e)] according to Eq. 1 [49].

First-order genetic diversity

1 $${\text{D}}_{{{\text{w}},{\text{p}}}} = { \exp }\left( { - \mathop \sum \limits_{{{\text{i}} = 1}}^{{{\text{R}}_{{{\text{w}},{\text{p}}}} }} {\text{p}}_{{{\text{i}}_{{{\text{w}},{\text{p}}}} }} { \ln }\left[ {{\text{p}}_{{{\text{i}}_{{{\text{w}},{\text{p}}}} }} } \right]} \right)$$

Diversity, *D*, weighs the abundance of all variants, *p*, at a given position, *i*, in the protein. A window length, *w*, is applied, with *w* = 1 used in order to independently assess the diversity of each position within a multiple sequence alignment. At an order, or Hill number, of q = 1, *D* does not exist; however, the limit as *q* approaches 1 can be computed as shown here.

Diversity at order = 1 calculates the effective number of species (amino acids or nucleotides) in a population while giving greater weight to neither rare nor abundant species. The maximum possible diversity is 20 for amino acid sequences (gp120 and Tat) and 4 for nucleotide sequences (LTR), with gaps regarded as missing data. In general, positions of high structural or functional importance are evolutionarily constrained in their use of amino acids or nucleotides and therefore demonstrate low diversity, while positions more permissive to variation in amino acid or nucleotide usage displayed higher genetic diversity [50]. Rarefaction curves for each position were generated in order to ensure that sufficient sample sizes existed for each comparison being made.

### Jensen–Shannon divergence

Jensen–Shannon divergence is a measure of the similarity between two probability distributions that can be applied to profile-to-profile multiple sequence alignment comparisons, with the divergence score bound by 0 (similar) and 1 (dissimilar) [51, 52]. Multiple sequence alignments (MSA) generated from R5- and X4-classified sequence populations were used to generate position frequency matrices (PFMs). Each PFM contains the relative abundance of each residue (amino acid or nucleotide) for each position (N) of the multiple sequence alignment, resulting in 20 × N or 4 × N matrices for amino acid or nucleotide sequences, respectively. Residues that are not present in any of the sequences at a particular position of the MSA were represented with a pseudo-count of 1 × 10^−7^, equivalent to a relative abundance of 1 instance per ten million sequences, which ranges from approximately 1 × 10^4^-fold to 1 × 10^6^-fold lower abundance than being present in a single sequence. PFMs derived from R5- and X4-classified sequences were used to calculate the Jensen–Shannon divergence between populations according to Eq. 2.

Jensen–Shannon divergence

2 $${\text{D}}_{\text{JS}} = \frac{1}{2}\left[ {\mathop \sum \limits_{{{\text{a}} = 1}}^{20} {\text{Q}}_{\text{a}}^{1} { \log }_{2} \frac{{{\text{Q}}_{\text{a}}^{1} }}{{{\text{Q}}_{\text{a}}^{0} }} + \mathop \sum \limits_{{{\text{a}} = 1}}^{20} {\text{Q}}_{\text{a}}^{2} { \log }_{2} \frac{{{\text{Q}}_{\text{a}}^{2} }}{{{\text{Q}}_{\text{a}}^{0} }} } \right]$$

where

$${\text{Q}}_{\text{a}}^{0} = \frac{1}{2}\left( {{\text{Q}}_{\text{a}}^{1} + {\text{Q}}_{\text{a}}^{2} } \right)$$

Jensen–Shannon divergence, *D*_*JS*_, is determined according to the abundance of each amino acid variant, *Q*_*a*_, in populations 1 and 2, using an information theory-based calculation. The value *Q*^0^ is calculated for each amino acid variant, and a pseudo-count is utilized for amino acid variants absent in both populations.

Statistically significant positions were identified by applying a Monte Carlo permutation test, which randomly re-grouped the total pool of sequences into groups of size M and N iteratively (n = 1000), where M and N are equivalent to the number of sequences in the X4 and R5 groups, and generated a probability density function (PDF) of the Jensen–Shannon divergence values of the randomized model using a Gaussian kernel density estimator implemented in SciPy. Numerical integration was used to determine the probability of finding a random value greater than or equal to the true Jensen–Shannon divergence.

### Statistical analysis

Statistical analysis was performed in custom IPython Notebooks using the SciPy Python library (version 0.14.0). Spearman’s rank correlation coefficient was used to evaluate the relationship between R5 and X4 diversity (Fig. 1), as well as the relationship of Jensen–Shannon divergence to mean genetic diversity and the absolute difference in genetic diversity (Fig. 2), respectively. gp120 domain enrichment analysis (Fig. 4) of statistically divergent positions utilized a hypergeometric test, with a null hypothesis of equal distribution of divergent positions. Enrichment of consensus amino acids within statistically divergent Tat positions was performed using a Fisher’s exact test (Fig. 6).

### Two sequence logos

Experimentally validated transcription factor bindings sites C/EBP-II (HXB2 positions 281–289), ATF-CREB (330–337), C/EBP-I (338–349), NF-κB-II (350–359), NF-κB-I (363–373), Sp-III (377–386), Sp-II (388–398), and Sp-I (399–408), as well as the RNA stem loop (454–518), were evaluated using *Two Sample Logo* [53]. *Two Sample Logo* is a web-based application that calculates and visualizes the differences between two sets of aligned sequences. Each nucleotide was represented with a different color, and the height of the one-letter nucleotide code was scaled according to the magnitude of the difference in abundance of the nucleotide at a given position, with the largest difference in each comparison represented by the maximum height in the logo.

### Identification of putative transcription binding sites

Position frequency matrices (PFMs) were downloaded from the JASPAR redundant vertebrate database for C/EBP, Sp, NFκB, and CREB. Each PFM was converted into a position weight matrix (PWM) as previously described [52]. Each LTR sequence was scanned along its entirety to score every potential binding site using each of the PWMs. Each score was then compared to the maximum possible score for the PWM being used in order to determine a percentile score. Only binding sites with a mean percentile score >0.30 in either the R5- or X4-classified LTR sequences were considered for statistical analysis (Table 3). Binding affinities as defined by PWM score show a non-Gaussian distribution (data not shown). As such, the Kolmogorov–Smirnov (KS) test was used to compare affinities between different groups. The PWM was applied to each LTR and then the R5 and X4 distributions were compared. The P values were adjusted using the Benjamini–Hochberg procedure.
